# Supplementary material for: Metal oxide-based gas sensor array for VOCs determination in complex mixtures using machine learning
Source: Mikrochim Acta. 2024 Mar 13;191(4):196. doi: 10.1007/s00604-024-06258-8 (PMC10937778; doi:10.1007/s00604-024-06258-8)
Supplement: Supplementary file 1 — Supplementary file1 (DOCX 6747 KB) [file 604_2024_6258_MOESM1_ESM.docx]

**Online Resource**

**Metal oxide-based gas sensor array for VOCs determination in complex mixtures using machine learning**

**Shivam Singh^1^, Sajana S^1^,** **Poornima Varma^2^**, **Gajje Sreelekha^3^**, **Chandranath Adak^3,*^, Rajendra P. Shukla^4,*^, Vinayak B. Kamble^1,*^**

^1^School of Physics, Indian Institute of Science Education and Research Thiruvananthapuram, 695551 India.

^2^Dept. of CSE, Indian Institute of Information Technology Lucknow, Uttar Pradesh 226002, India.

**^3^**Dept. of CSE, Indian Institute of Technology Patna, Bihar 801106, India.

^4^BIOS Lab-on-a-Chip Group, MESA+ Institute for Nanotechnology, Max Planck Center for Complex Fluid Dynamics, University of Twente, P.O. Box 217, 7500 AE Enschede, The Netherlands.

Corresponding authors: Chandranath Adak, Rajendra P. Shukla, Vinayak Kamble.

# Various Volatile organic Compounds in exhaled breath as biomarkers for diagnostics and our approach

Exhaled Breath includes several volatile chemicals, most present in minimal ppb concentrations. Therefore, the proportion of exogenous VOC can be used as a gauge for human biology and physiological health.

**Ethanol:** Numerous potential uses, including exhaled condition monitoring for the detection of smaller doses of ethanol, have lately gained considerable attention. Breath ethanol levels in a healthy individual are typically below 380 parts per billion. Nevertheless, this might increase to 2300 ppb in cases of alcoholism and a history of fatty liver[1-4].

**Acetone:** person who has elevated breath acetone (T2DM > 1.71 ppm, T1DM > 2.19 ppm (Type 1 diabetes mellitus (T1DM), an asymptomatic disease, is caused by the body's antibodies attacking and killing the beta cells that produce insulin in the pancreas. However, in type 2 diabetes mellitus (T2DM), the body generates inadequate quantities of insulin or becomes resistant, making it difficult to maintain normal blood sugar levels. It often develops in adults and correlates with lifestyle factors, including obesity and inactivity) may well go approximately 21 ppm[1-3]), which would ring alarming bells of diabetes. A biochemical disorder like diabetes mellitus affects over 400 million people worldwide[4] linked to such occurrences.

**Toluene:** a high concentration of toluene in a person's breath has been recognized as a potential biomarker linked to lung diseases[5-7]. In addition, toluene has also been found to be a low-level VOC in type I and type II diabetes. This implies that toluene can also serve as a significant indicator or diagnostic tool for identifying symptoms associated with diabetes.

**Chloroform:** In contrast to the others, chloroform belongs to a broader class of compounds called trihalomethanes are linked to diseases like lung cancer[8, 9] thyroid abnormalities[10], etc. Nevertheless, their concentration range is yet to be established. In humans, breathing indoor air or consuming substantial quantities of chloroform-containing liquids such as chlorinated water may cause fatal consequences[11, 12], and the same can be diagnosed by the presence of chloroform in breath[13].

In this study, we employed a gas sensor array based on targets that were sputtered with a Direct Current (DC) source to deposit the metal oxides CuO, NiO and ZnO. The responses of various analytes passing over it were recorded using four volatile compounds: ethanol, toluene, acetone, and chloroform. The initial phase was mixing a single analyte with synthetic air and recording the response (resistance vs. time) for each electrode at 200 °C. One electrode was utilized at a time. After that, an experiment was conducted using two gases simultaneously, with one analyte being kept constant at a specific concentration while the other was changed. There were twelve different potential combinations for the 200 °C parallel readings. Similarly, the reaction was measured while carefully purging three gases, with two maintaining constant and the third changing. The gas sensor array's electrical response was examined using ML techniques. We have used different ML algorithms to analyze the data from the Metal Oxide Semiconductor (MOS) sensor array and compared their performances for the simultaneous detection of four VOCs. The ML algorithm was used to perform two types of analysis: (i) *classification* to categorize the varying gas/ chemical and (ii) *regression* analysis to predict the concentration of the gas. Therefore, not only qualitative but quantitative detection of four VOCs simultaneously allows the detection of multiple diseases and monitoring of the health of individuals. The proof-of-concept demonstration using a sensor array combined with ML algorithms can potentially analyze individual VOCs in breath samples to provide diagnostic and therapeutic information in diseases outlined such as lung cancer, heart diseases, diabetes and fibrosis, etc. Further miniaturization and its application to point-of-care testing devices can improve diagnostics and treatment monitoring of diseases (e.g., cancer).

# Thin film deposition details.

Table S1. Sputtering parameter and deposition conditions for all three oxides.

| **Deposition conditions of**  **The oxide film** | **CuO** | **NiO** | **ZnO** |
| --- | --- | --- | --- |
| Base Pressure(mbar) | 9.80E-6 | 3.84E-6 | 7.80E-6 |
| Deposition pressure(mbar) | 2.42E-2 | 2.59E-2 | 5.10E-2 |
| Target (1 inch diameter) | Cu metal (3 mm thickness) | Ni foil (1 mm thickness) | Zn metal (3 mm thickness) |
| Argon flow rate (SCCM) | 30 | 30 | 30 |
| Oxygen flow rate (SCCM) | 10 | 10 | 10 |
| Substrate | Alumina + Glass | Alumina + Glass | Alumina + Glass |
| Voltage applied | 538 V | 343 V | 377 V |
| Current | 0.08 A | 0.26 A | 0.05 A |
| Deposition time (min) | 14 | 25 | 25 |
| Substrate temperature | RT | RT | RT |
| Rotation used | Yes | Yes | Yes |

Table S1 summarizes the DC magnetron deposition parameters of the sensor material thin film fabrication.

# Gas sensor device Fabrication and dimensions

***
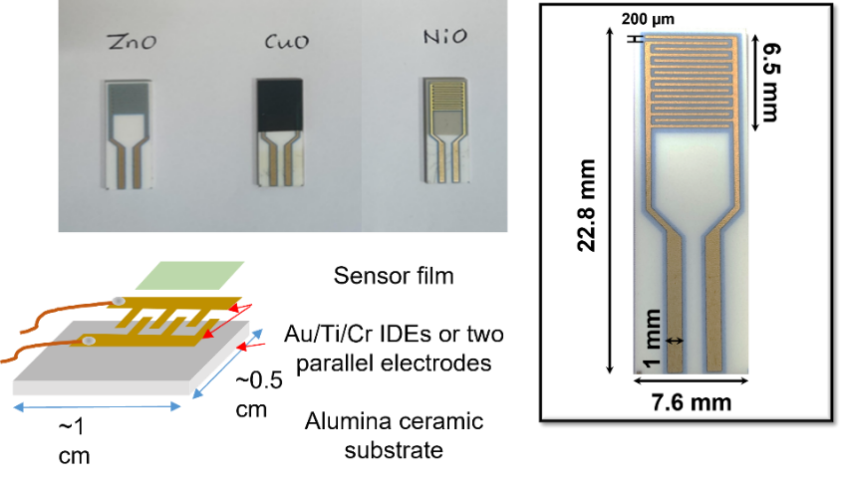
***

**ESM 1: The digital picture of the fabricated ZnO, CuO, and NiO sensors. The schematic diagram of the electrodes is shown in the bottom figure. The dimensions of the substrate and electrodes are shown in the adjacent figure.**

The electrode (Au) metal choice is crucial for the sensor response as a non-ohmic contact may be formed if the work functions alignment does not favor the bidirectional flow of current. A sensor design features an alumina substrate with interdigitated gold electrodes (IDEs). Here, to maintain the reproducibility of the nature of contact so that the data is consistent in repeated measurements, we chose prefabricated Au IDEs on the alumina substrates commercially available (by Metrohm India Pvt Ltd.).

The dimension is L 22.8 mm x W 7.6 mm x H 1 mm. The width and gap between the two electrodes are 200 µm.

# Gas sensing system details and design.

The VOCs were generated using bubblers and vapor pressure data-based calculations were employed to estimate concentrations.

Synthetic air was bubbled over volatile organic compounds kept at a constant cooling of zero degrees Celsius by an MFC-controlled carrier gas flow to produce the test gas vapors. Since the vapor pressure of the analyte can be determined at a given temperature using the Antoine equation, it is possible to calculate the gas concentration by equilibrating it to the environment. Eq (S1) passes the attention;

To create the test gas vapors, synthetic air was bubbled over volatile organic compounds maintained at a constant cooling of zero degrees Celsius by an MFC-controlled carrier gas flow. Since gas concentration can be calculated by equilibrating the vapor pressure of the analyte to the atmosphere, as the vapor pressure of the VOCs is known at a given temperature. The concentration is given by eq;

$C\left( ppm \right)=\frac{F_{s}}{F_{s}+F_{c}+F_{D}}\times{10}^{6}$ (S1)

where, F_d_ is the dilution gas flow rate in SCCM, F_c_ is the input flow rate to the analyte in SCCM, and F_s_ is the output flow rate of the vapor and can be calculated as shown in eq.

$F_{s}= \left( \frac{P_{th}}{P_{o}{-P}_{th}} \right)F_{c}$ (S2)

Here, P_th_ is the thermodynamic vapor pressure of the analyte at that temperature, and P_o_ is the atmospheric vapor pressure. More details can be found elsewhere[14, 15].

As mentioned in Section 2.1.4, the volatile liquids are used to generate the vapors of the desired gas for sensing. Here, the bubblers are maintained at a constant temperature, and the carrier gas is bubbled through the liquid in the thermostat to generate the vapors subjected to sensor exposure. Here, the concentrations of the vapors are mainly governed by constant temperature baths and the flow rate of the carrier gas to a certain extent. Therefore, the gas concentrations utilized were primarily governed by the generation rate and vapor pressure. The primary objective was to explore and investigate the region where the concentrations of these interfering biomarkers were high. Subsequently, the sensor response was recorded by introducing variable gas concentrations within this specific range, as mentioned above. This approach thoroughly examines and characterizes the sensor's behavior when exposed to various interfering gases at different concentrations. Besides, the gas sensing apparatus' practical limits, such as MFC resolution accuracy, primarily determined our study's interference gas concentration selection. We have been focusing on our system's capacity to manage intricate gas combinations while optimizing and miniaturizing them. Working with metal oxides has been a critical component of our strategy since it allows us to extrapolate response concentration curves to lower parts per million (ppm) concentrations. Tapping into the power of machine learning to improve our system's accuracy and forecasting powers at these lower concentrations is envisaged—the strategy to be rigorously trained and fine-tuned to produce highly accurate predictions even at sub-ppm levels.

# Combinations of gases undertaken for sensor array response characteristics

Table S2: Combinations of VOCs used for 2-gases and 3-gases experiments.

| **Variable VOC** | **2-gases**  **(Constant VOCs)** | **3-gases**  **(Constant VOCs)** |
| --- | --- | --- |
|  |  |  |
| **Ethanol** | Acetone (2000 ppm) | Chloroform (1000 ppm) + Toluene (720 ppm) |
|  | Toluene (1880 ppm) | Acetone (600 ppm) + Chloroform (1000 ppm) |
|  | Chloroform (1880 ppm) | Acetone (600 ppm) + Toluene (720 ppm) |
| **Acetone** | Toluene (1880 ppm) | Ethanol (900 ppm) + Toluene (540 ppm) |
|  | Chloroform (1880 ppm) | Chloroform (1000 ppm) + Toluene (720 ppm) |
|  | Ethanol (2400 ppm) | Chloroform (1000 ppm) + Ethanol (900 ppm) |
| **Toluene** | Chloroform (1880 ppm) | Acetone (1000 ppm) + Ethanol (900 ppm) |
|  | Ethanol (1880ppm) | Ethanol (900 ppm) + Chloroform (1000 ppm) |
|  | Acetone (2000 ppm) | Acetone (600 ppm) + Chloroform (1000 ppm) |
| **Chloroform** | Ethanol (2400 ppm) | Acetone (600 ppm) + Ethanol (900 ppm) |
|  | Acetone (1600 ppm) | Ethanol (900 ppm) + Toluene (720 ppm) |
|  | Toluene (1000 ppm) | Acetone (400 ppm) + Toluene (540 ppm) |

The various combinations of gases tested for generating the test data-set and training the algorithms have been described in Table S2.

# The correlation matrices of the various features for each of the gas mixture studied have been shown in ESM 2 below.


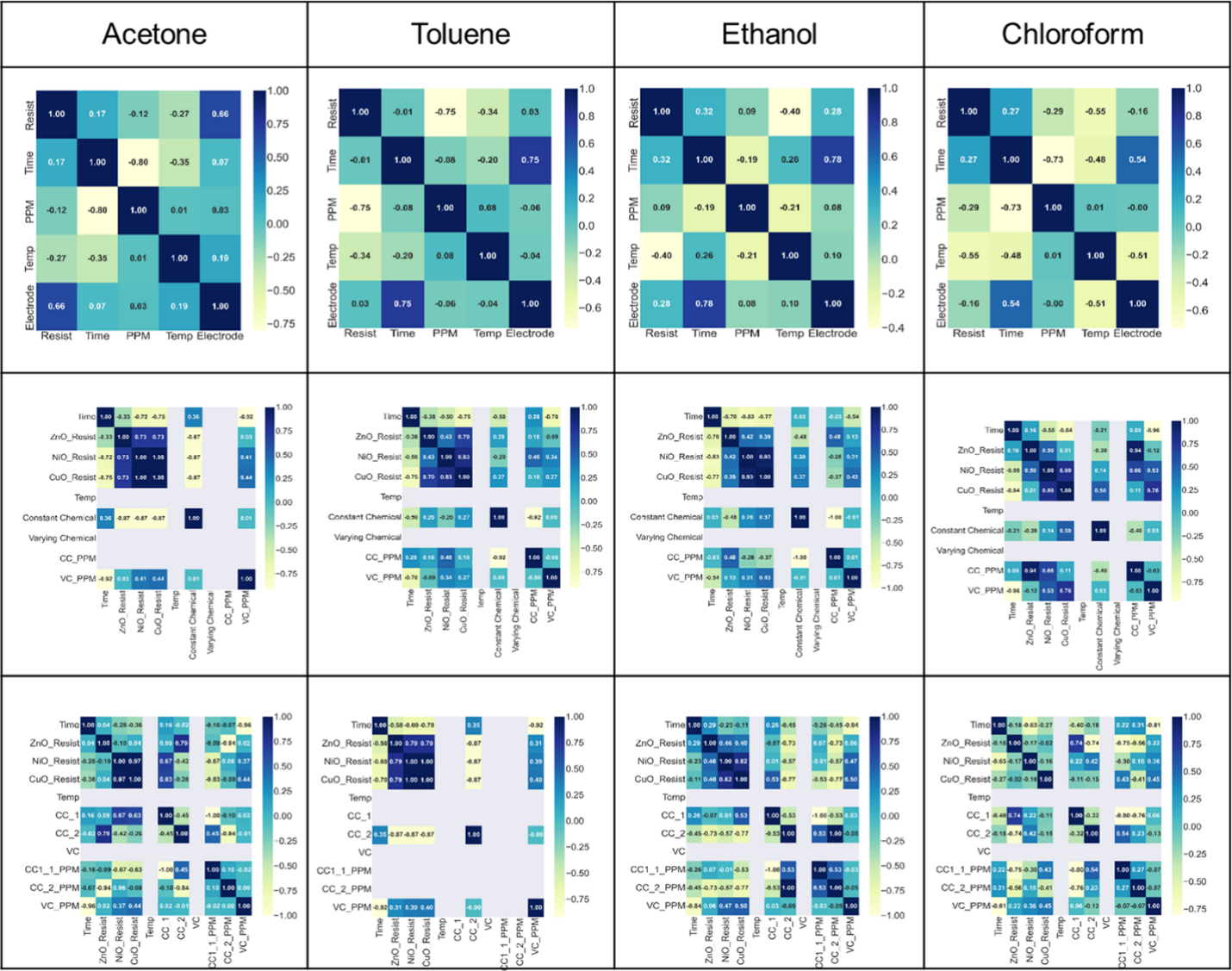


ESM 2: Correlation matrices: 1^st^ row: 1-gas dataset, 2^nd^ row: 2-gases dataset, 3^rd^ row: 3-gases dataset.

ESM 3: The Energy dispersive spectra showing compositions of (a) CuO, (b) NiO, and (c) ZnO thin films. (d) the SEM image of the Au interdigitated electrodes with oxide film deposited on top. (e) the digital photograph of the device to locate the region in the image (d) and (f-k).


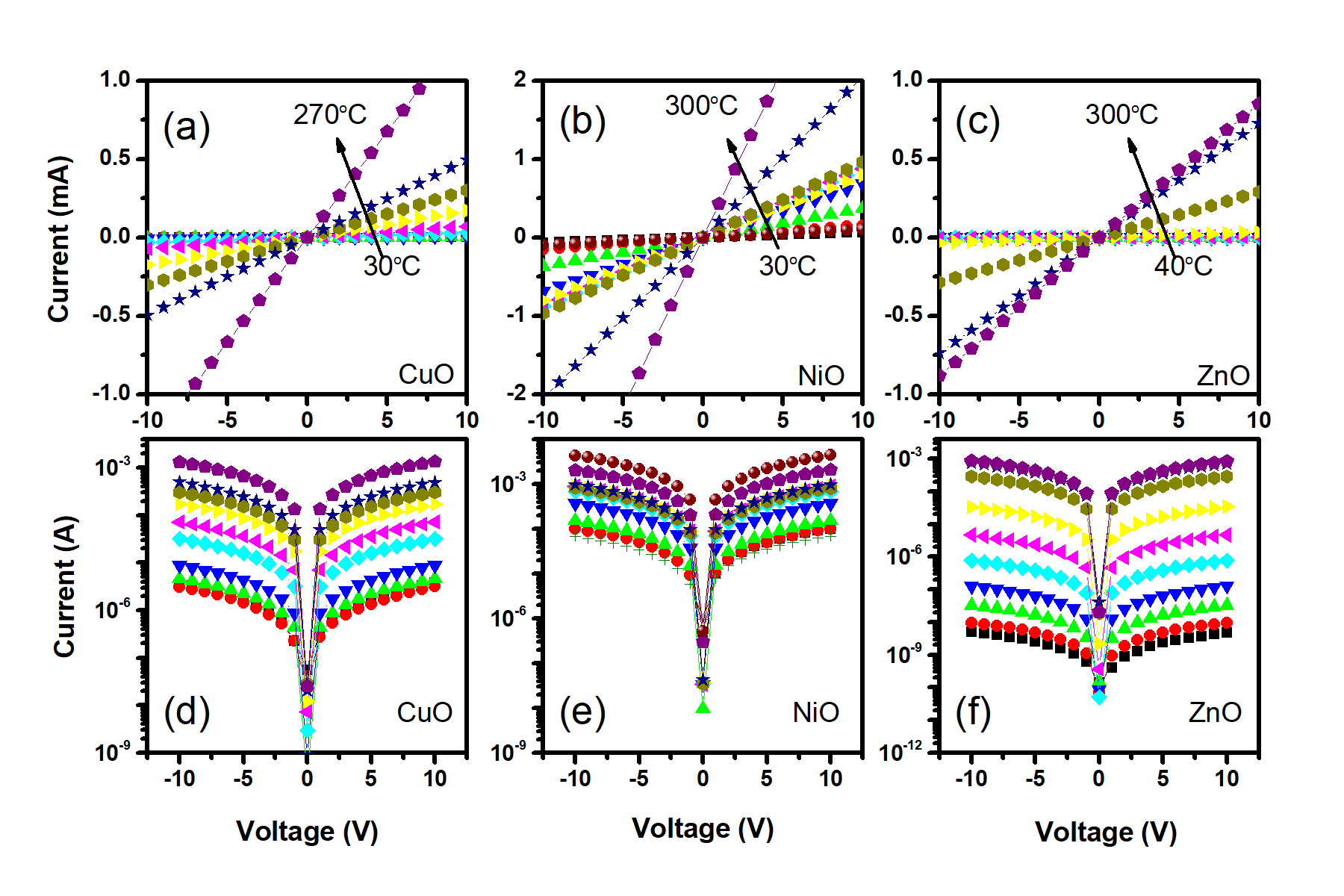


ESM 4: Current vs voltage plot for (a, d) CuO, (b,e) NiO and (c,f) ZnO at different temperatures in linear (top row) and log scale (bottom row).

The nature of the contact (i.e. ohmic or schottky) were tested before performing the sensing studies by measuring I-V characteristics. The same are shown in ESM 4. The IVs have been found to be linear implying an ohmic contact. Besides, their temperature dependence is studied by taking IV at each temperature interval of 25 degrees from room temperature to 300 degrees.


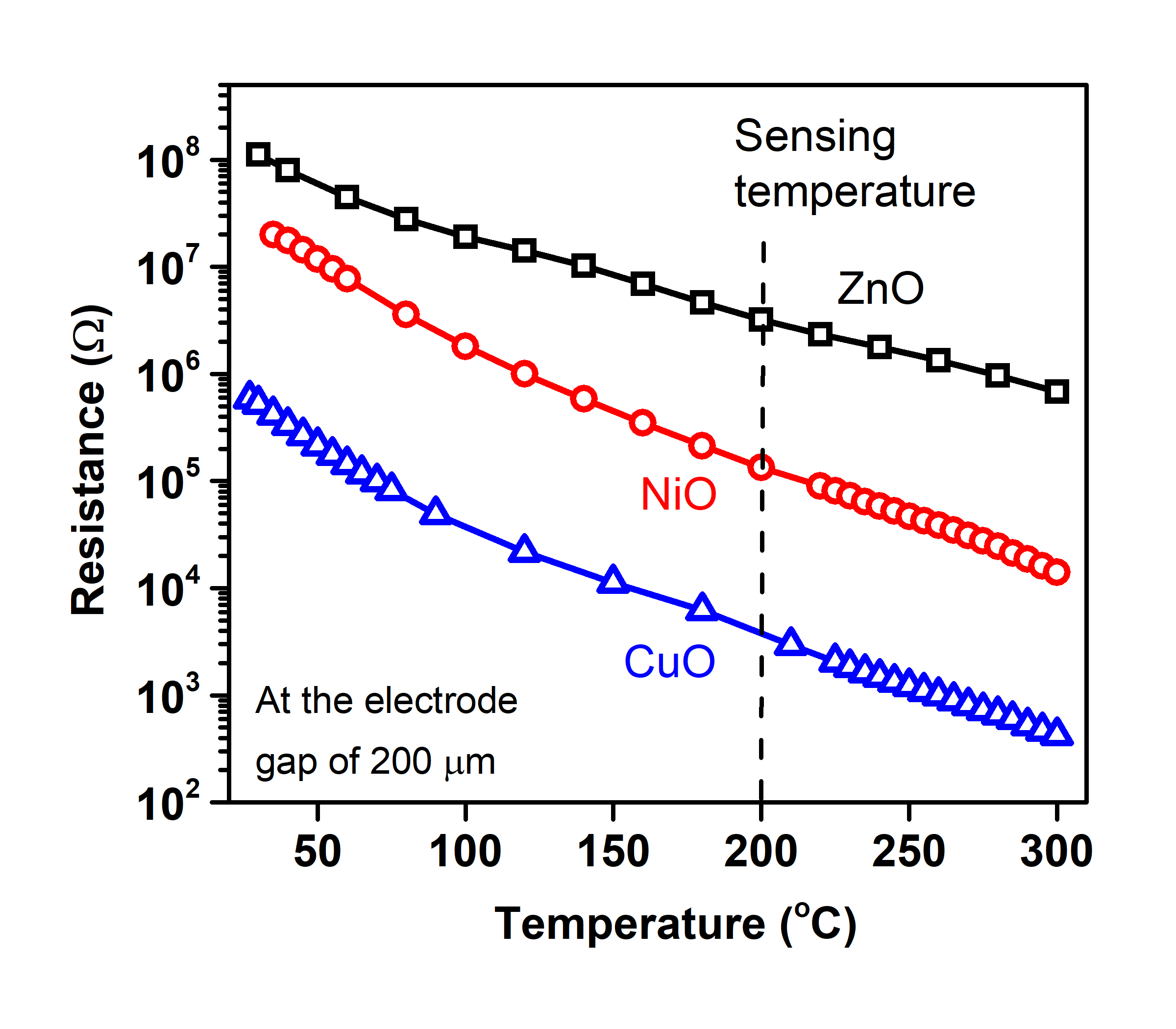


ESM 5: The Resistance vs. Temperature plot for all three metal oxides.

ESM 5: show the resistance vs temperature data of all the three sensor materials studied. They show a semiconducting behavior as expected. Among all the three sensors ZnO is the highest resistance sample. Nevertheless, at operating conditions of 200 ᵒC all the three resistance are below 10 MΩ value which is suitable for the electronics.

**Gas sensing studies.**

The gas response for each of the studies undertaken to study the response of single, double, and 3-gas simultaneously have been shown below. The data shown here has been used for the analysis mentioned in the main text.
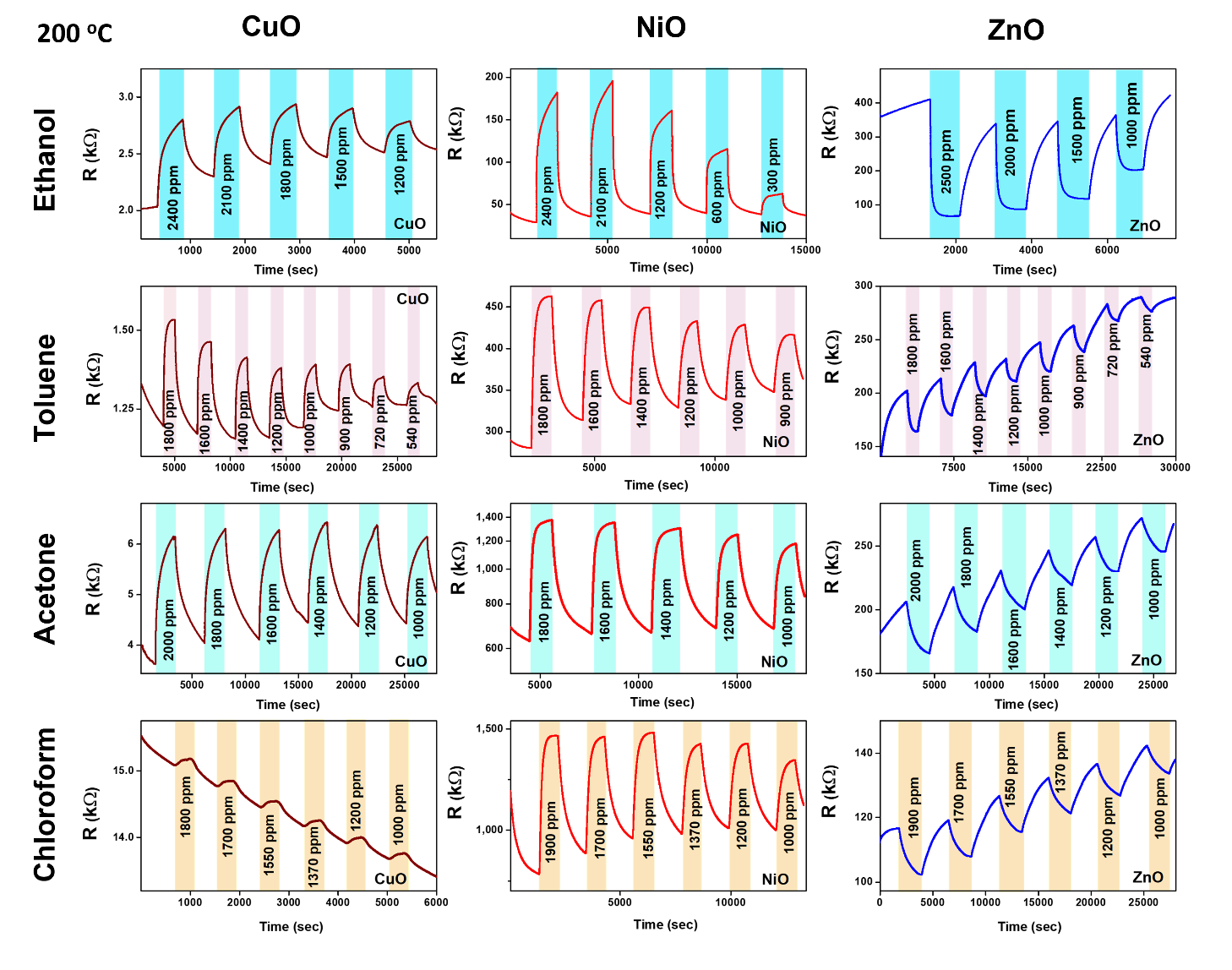


ESM 6: The Resistance vs time plots of CuO, NiO and ZnO for Ethanol, Toluene, Acetone, and Chloroform respectively. (Single gas)


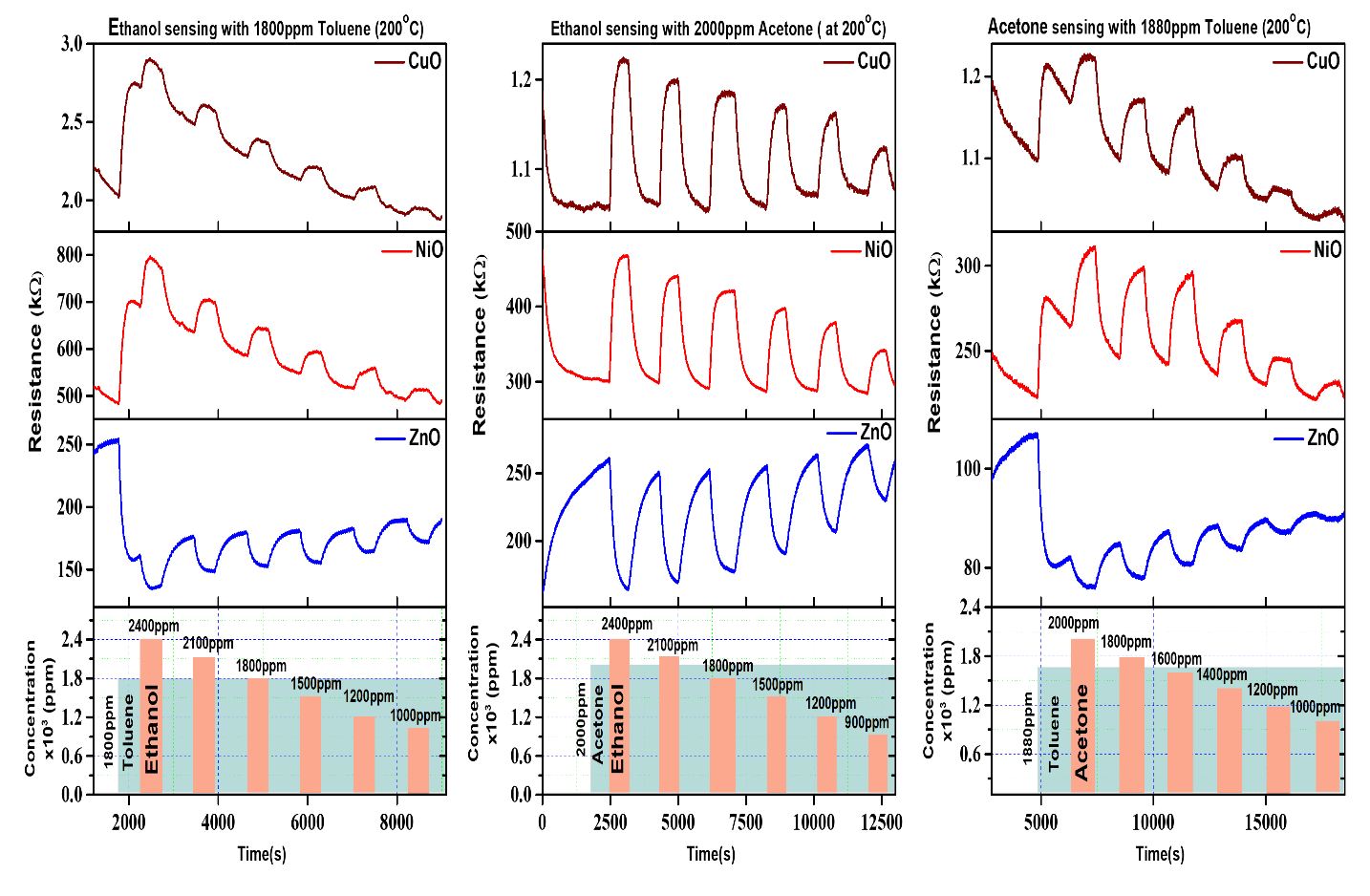


ESM 7: The double gas response was studied with one stationary and one variable gas. (Two gas)


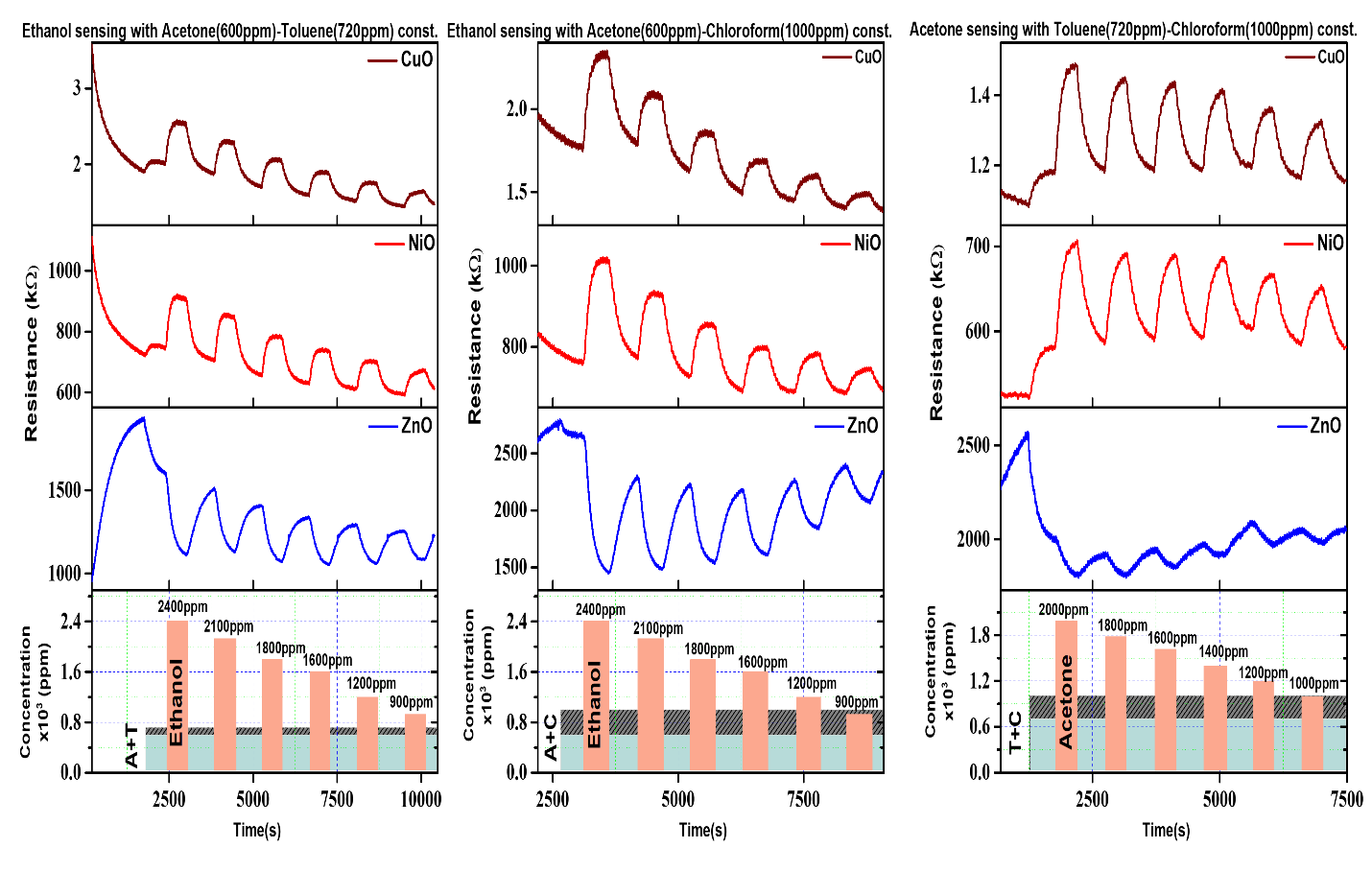
ESM 8. The response to ethanol vapors in presence of mixtures of toluene, acetone and chloroforms. (three gas)

The gas sensor response of CuO and ZnO for all the four gases individually as well as with the interfering species has been studied and the same is shown in ESM 8. It may be seen that the response is significantly different when measured individually vis-à-vis in presence of other gases.


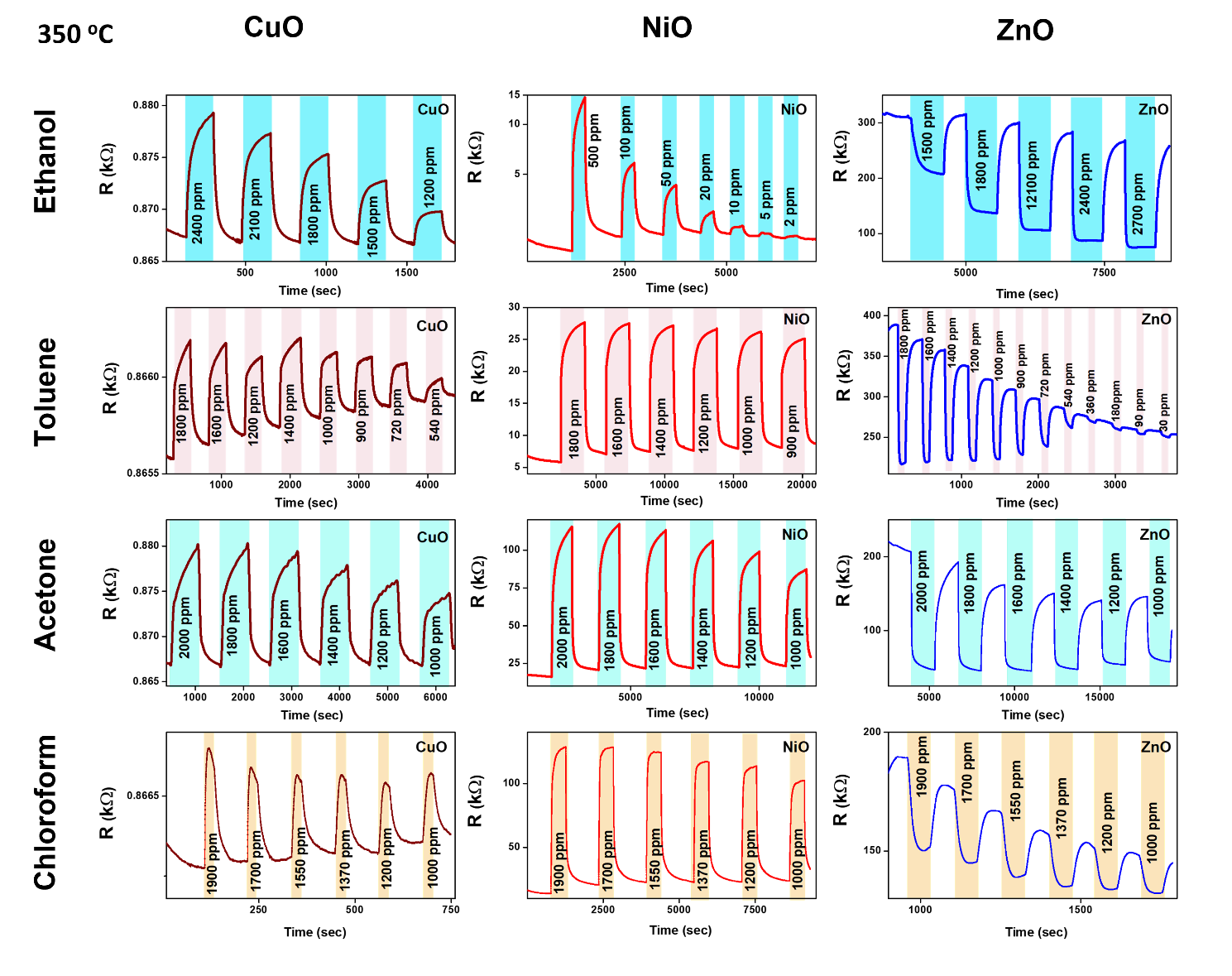


ESM 9: The Resistance vs. time plots of CuO, NiO, and ZnO for Ethanol, Toluene, Acetone, and Chloroform, respectively, at 350^o^C. (Single gas)

Table S3: Variances of first 5 principal components (PC) over various gas mixture datasets

| **Variability (%)** | **PC**  **Dataset** | **PC1** | **PC2** | **PC3** | **PC4** | **PC5** |
| --- | --- | --- | --- | --- | --- | --- |
|  | **1-gas** | 38.81186 | 28.86281 | 20.33076 | 8.01912 | 3.97542 |
|  | **2-gases** | 41.81045 | 23.51523 | 15.22549 | 8.58879 | 7.50794 |
|  | **3-gases** | 34.83254 | 23.81192 | 17.54433 | 10.54873 | 5.85302 |


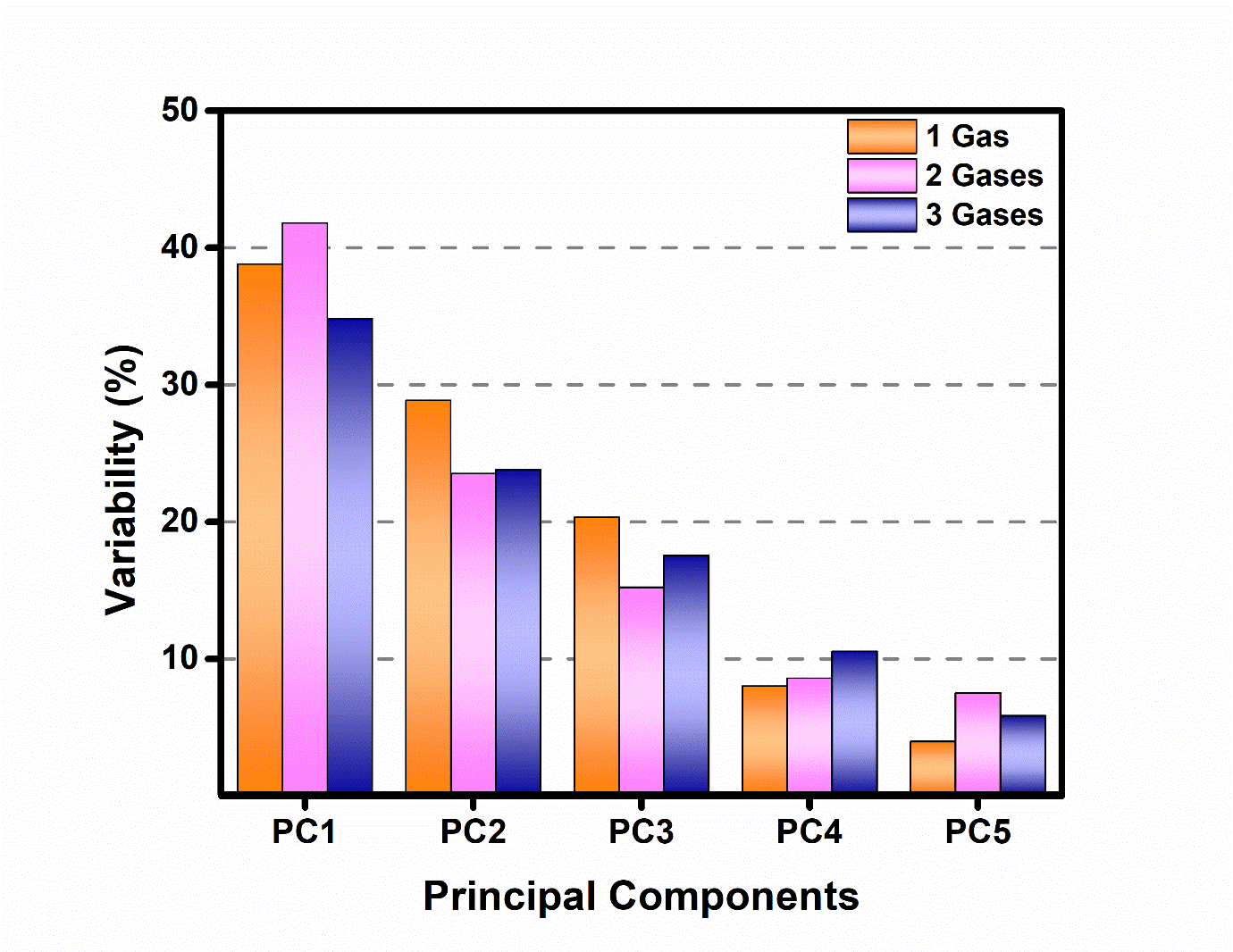


ESM 10: Pictorial representation of variability of first 5 principal components (PC) over various gas mixture datasets

To reduce the complexity of the data while preserving trends and patterns, we used Principal Component Analysis (PCA) on the sensor signal response. The variances of first 5 principal components (PC1, PC2, PC3, PC4, and PC5) are shown in Table S3 for 1-gas, 2-gases, and 3-gases datasets, and also represented pictorially in Fig. S11.

Considering PC1 and PC2, we obtained the 2D plots of ESM 11 and ESM 12 over 1-gas and 2-gases datasets, respectively.


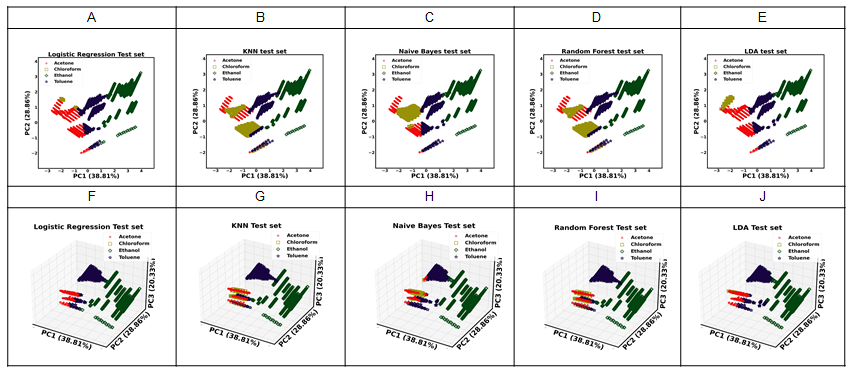


ESM 11: 1-gas dataset: 2D and 3D classification plots in 1^st^ and 2^nd^ rows, respectively.


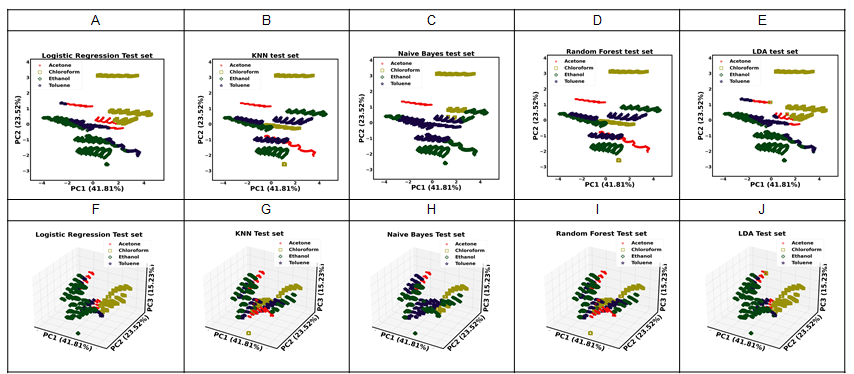


ESM 12: 2-gases dataset: 2D and 3D classification plots in 1^st^ and 2^nd^ rows, respectively.

Tables S4, S5, and S6 compare the experimental results obtained on 1-gas, 2-gases, and 3-gases datasets using KNN regression, ANN, random forest, decision tree, and linear regression models.

Table S4: Applying various machine learning model 1-gas dataset

| **Method** | **Gas Name** | **RMSE** | **MSE** | **MAE** | **NRMSE** | **R^2^** | **LoD** | **LoQ** |
| --- | --- | --- | --- | --- | --- | --- | --- | --- |
| **KNN Regression** | Acetone | 0.00086 | 7.43×10^-7^ | 0.00001 | 0.00114 | 0.99997 | 0.00344 | 0.01146 |
|  | Toluene | 0.00082 | 6.77×10^-7^ | 0.00001 | 0.00109 | 0.99997 | 0.00328 | 0.01095 |
|  | Ethanol | 0.00076 | 5.82×10^-7^ | 0.00001 | 0.00101 | 0.99997 | 0.00304 | 0.01015 |
|  | Chloroform | 0.00153 | 2.35×10^-6^ | 0.00004 | 0.00203 | 0.99990 | 0.00611 | 0.02039 |
| **ANN** | Acetone | 0.23798 | 0.05663 | 0.17747 | 0.58080 | 0.43819 | 1.83359 | 6.11199 |
|  | Toluene | 0.37517 | 0.14075 | 0.30339 | 0.67179 | 0.00000 | 6.86907 | 22.89691 |
|  | Ethanol | 0.46381 | 0.21512 | 0.37303 | 0.82863 | 0.00000 | 7.55353 | 25.17844 |
|  | Chloroform | 0.02368 | 0.00056 | 0.00787 | 0.04346 | 0.99435 | 0.13167 | 0.43890 |
| **Random Forest** | Acetone | 0.01235 | 0.00015 | 0.00248 | 0.01645 | 0.99412 | 0.05032 | 0.16774 |
|  | Toluene | 0.01300 | 0.00016 | 0.00265 | 0.01730 | 0.99348 | 0.05299 | 0.17663 |
|  | Ethanol | 0.01218 | 0.00014 | 0.00233 | 0.01621 | 0.99426 | 0.04955 | 0.16519 |
|  | Chloroform | 0.01215 | 0.00014 | 0.00233 | 0.01618 | 0.99428 | 0.04945 | 0.16484 |
| **Decision Tree** | Acetone | 0.08577 | 0.00735 | 0.06750 | 0.11416 | 0.71512 | 0.47851 | 1.59505 |
|  | Toluene | 0.08602 | 0.00739 | 0.06766 | 0.11452 | 0.71391 | 0.48104 | 1.60349 |
|  | Ethanol | 0.08589 | 0.00737 | 0.06761 | 0.11437 | 0.71547 | 0.47996 | 1.59987 |
|  | Chloroform | 0.08596 | 0.00738 | 0.06762 | 0.11444 | 0.71526 | 0.48053 | 1.60176 |
| **Linear Regression** | Acetone | 0.06823 | 0.00465 | 0.05018 | 0.09082 | 0.82034 | 0.33194 | 1.10646 |
|  | Toluene | 0.06830 | 0.00466 | 0.05022 | 0.09095 | 0.81937 | 0.33265 | 1.10883 |
|  | Ethanol | 0.06839 | 0.00467 | 0.05033 | 0.09102 | 0.81945 | 0.33327 | 1.11091 |
|  | Chloroform | 0.06827 | 0.00466 | 0.05025 | 0.09087 | 0.82019 | 0.33250 | 1.10833 |

Table S5: Applying various machine learning model 2-gases dataset

| **Method** | **Gas Name** | **RMSE** | **MSE** | **MAE** | **NRMSE** | **R^2^** | **LoD** | **LoQ** |
| --- | --- | --- | --- | --- | --- | --- | --- | --- |
| **KNN** | Acetone | 0.00131 | 1.72×10^-6^ | 0.00002 | 0.00319 | 0.99996 | 0.00957 | 0.03190 |
|  | Toluene | 0.00094 | 8.98×10^-7^ | 0.00001 | 0.00226 | 0.99998 | 0.00678 | 0.02260 |
|  | Ethanol | 0.00095 | 9.21×10^-7^ | 0.00001 | 0.00230 | 0.99998 | 0.00692 | 0.02309 |
|  | Chloroform | 0.00194 | 3.79×10^-6^ | 0.00006 | 0.00466 | 0.99992 | 0.01400 | 0.04669 |
| **ANN** | Acetone | 0.29005 | 0.08413 | 0.18941 | 0.74214 | 0.24191 | 4.13273 | 13.77579 |
|  | Toluene | 0.26750 | 0.07155 | 0.22071 | 0.70174 | 0.39794 | 3.74026 | 12.46753 |
|  | Ethanol | 0.29169 | 0.08508 | 0.23761 | 0.61701 | 0.21728 | 4.07928 | 13.59760 |
|  | Chloroform | 0.01832 | 0.00033 | 0.00732 | 0.03550 | 0.99656 | 0.10766 | 0.35887 |
| **Random Forest** | Acetone | 0.00197 | 3.89×10^-6^ | 0.00018 | 0.00476 | 0.99992 | 0.01429 | 0.04766 |
|  | Toluene | 0.00182 | 3.33×10^-6^ | 0.00017 | 0.00437 | 0.99993 | 0.01313 | 0.04378 |
|  | Ethanol | 0.00108 | 1.18×10^-6^ | 0.00009 | 0.00260 | 0.99997 | 0.00782 | 0.02608 |
|  | Chloroform | 0.00182 | 3.35×10^-6^ | 0.00018 | 0.00441 | 0.99993 | 0.0132 | 0.04414 |
| **Decision Tree** | Acetone | 0.07637 | 0.00583 | 0.06633 | 0.18478 | 0.88192 | 0.62754 | 2.09183 |
|  | Toluene | 0.07653 | 0.00585 | 0.06650 | 0.18394 | 0.88135 | 0.62767 | 2.09226 |
|  | Ethanol | 0.07667 | 0.00587 | 0.06653 | 0.18448 | 0.87986 | 0.62808 | 2.09362 |
|  | Chloroform | 0.07677 | 0.00589 | 0.06683 | 0.18403 | 0.88072 | 0.62697 | 2.08992 |
| **Linear Regression** | Acetone | 0.03360 | 0.00112 | 0.02753 | 0.08087 | 0.97698 | 0.24889 | 0.82965 |
|  | Toluene | 0.03379 | 0.00114 | 0.02768 | 0.08132 | 0.97672 | 0.24978 | 0.83262 |
|  | Ethanol | 0.03351 | 0.00112 | 0.02747 | 0.08055 | 0.97710 | 0.24709 | 0.82363 |
|  | Chloroform | 0.03366 | 0.00113 | 0.02756 | 0.08109 | 0.97695 | 0.24957 | 0.83193 |

Table S6: Applying various machine learning model 3-gases dataset

| **Method** | **Gas Name** | **RMSE** | **MSE** | **MAE** | **NRMSE** | **R^2^** | **LoD** | **LoQ** |
| --- | --- | --- | --- | --- | --- | --- | --- | --- |
| **KNN** | Acetone | 0.00163 | 2.67×10^-6^ | 0.00005 | 0.00393 | 0.99994 | 0.01179 | 0.03932 |
|  | Toluene | 0.00204 | 4.19×10^-6^ | 0.00006 | 0.00496 | 0.99991 | 0.01488 | 0.04961 |
|  | Ethanol | 0.00196 | 3.87×10^-6^ | 0.00005 | 0.00474 | 0.99992 | 0.01422 | 0.04742 |
|  | Chloroform | 0.00342 | 1.17×10^-5^ | 0.00020 | 0.00825 | 0.99976 | 0.02478 | 0.08260 |
| **ANN** | Acetone | 0.12812 | 0.01641 | 0.10564 | 0.23949 | 0.85374 | 0.82988 | 2.76628 |
|  | Toluene | 0.25066 | 0.06283 | 0.19068 | 0.64111 | 0.48192 | 2.54640 | 8.48802 |
|  | Ethanol | 0.18948 | 0.03590 | 0.15538 | 0.32139 | 0.66963 | 1.31273 | 4.37577 |
|  | Chloroform | 0.03286 | 0.00108 | 0.01765 | 0.05478 | 0.98816 | 0.16329 | 0.54430 |
| **Random Forest** | Acetone | 0.00365 | 1.34×10^-5^ | 0.00016 | 0.00883 | 0.99972 | 0.02650 | 0.08836 |
|  | Toluene | 0.00273 | 7.46×10^-6^ | 0.00010 | 0.00661 | 0.99984 | 0.01985 | 0.06616 |
|  | Ethanol | 0.00227 | 5.19×10^-6^ | 0.00007 | 0.00548 | 0.99989 | 0.01646 | 0.05488 |
|  | Chloroform | 0.00369 | 1.36×10^-5^ | 0.00016 | 0.00882 | 0.99972 | 0.02648 | 0.08828 |
| **Decision Tree** | Acetone | 0.05921 | 0.00350 | 0.04852 | 0.14292 | 0.92822 | 0.46004 | 1.53348 |
|  | Toluene | 0.05986 | 0.00358 | 0.04902 | 0.14489 | 0.92787 | 0.46872 | 1.56241 |
|  | Ethanol | 0.05983 | 0.00358 | 0.04903 | 0.14388 | 0.92846 | 0.46418 | 1.54728 |
|  | Chloroform | 0.06019 | 0.00362 | 0.04923 | 0.14590 | 0.92783 | 0.47167 | 1.57224 |
| **Linear Regression** | Acetone | 0.03552 | 0.00126 | 0.02762 | 0.08468 | 0.97455 | 0.26122 | 0.87076 |
|  | Toluene | 0.03508 | 0.00123 | 0.02728 | 0.08471 | 0.97506 | 0.25984 | 0.86614 |
|  | Ethanol | 0.03550 | 0.00126 | 0.02764 | 0.08538 | 0.97480 | 0.26317 | 0.87725 |
|  | Chloroform | 0.03531 | 0.00124 | 0.02743 | 0.08538 | 0.97501 | 0.26287 | 0.87623 |

Table S7: Comparison of R^2^ obtained by employed ML-based regression architectures

| **Dataset** | **Gas Name** | **KNN Regression** | **ANN** | **Random Forest** | **Decision Tree** | **Linear Regression** |
| --- | --- | --- | --- | --- | --- | --- |
| **1-gas** | Acetone | **0.99997** | 0.43819 | 0.99412 | 0.71512 | 0.82034 |
|  | Toluene | **0.99997** | 0.00000 | 0.99348 | 0.71391 | 0.81937 |
|  | Ethanol | **0.99997** | 0.00000 | 0.99426 | 0.71547 | 0.81945 |
|  | Chloroform | **0.99990** | 0.99435 | 0.99428 | 0.71526 | 0.82019 |
| **2-gases** | Acetone | **0.99996** | 0.24191 | 0.99992 | 0.88192 | 0.97698 |
|  | Toluene | **0.99998** | 0.39794 | 0.99993 | 0.88135 | 0.97672 |
|  | Ethanol | **0.99998** | 0.21728 | 0.99997 | 0.87986 | 0.97710 |
|  | Chloroform | 0.99992 | 0.99656 | **0.99993** | 0.88072 | 0.97695 |
| **3-gases** | Acetone | **0.99994** | 0.85374 | 0.99972 | 0.92822 | 0.97455 |
|  | Toluene | **0.99991** | 0.48192 | 0.99984 | 0.92787 | 0.97506 |
|  | Ethanol | **0.99992** | 0.66963 | 0.99989 | 0.92846 | 0.97480 |
|  | Chloroform | **0.99976** | 0.98816 | 0.99972 | 0.92783 | 0.97501 |

**References:**

[1] Deng C, Zhang J, Yu X, Zhang W and Zhang X (2004) Determination of acetone in human breath by gas chromatography-mass spectrometry and solid-phase microextraction with on-fiber derivatization Journal of chromatography. B, Analytical technologies in the biomedical and life sciences 810:269-75

[2] Wang C, Mbi A and Shepherd M (2010) A Study on Breath Acetone in Diabetic Patients Using a Cavity Ringdown Breath Analyzer: Exploring Correlations of Breath Acetone With Blood Glucose and Glycohemoglobin A1C IEEE Sensors Journal 10:54-63

[3] Turner C, Walton C, Hoashi S and Evans M (2009) Breath acetone concentration decreases with blood glucose concentration in type I diabetes mellitus patients during hypoglycaemic clamps Journal of breath research 3:046004

[4] Xiling L, Xu Y, Pan X, Xu J, Ding Y, Sun X, Song X, Ren Y and Shan P-F (2020) Global, regional, and national burden and trend of diabetes in 195 countries and territories: an analysis from 1990 to 2025 Scientific reports 10:14790

[5] Boots A W, Smolinska A, van Berkel J J B N, Fijten R R R, Stobberingh E E, Boumans M L L, Moonen E J, Wouters E F M, Dallinga J W and Van Schooten F J (2014) Identification of microorganisms based on headspace analysis of volatile organic compounds by gas chromatography–mass spectrometry Journal of Breath Research 8:027106

[6] Szulejko J E, McCulloch M, Jackson J, McKee D L, Walker J C and Solouki T (2010) Evidence for Cancer Biomarkers in Exhaled Breath IEEE Sensors Journal 10:185-210

[7] Ahmed A M, Mehaney A and Elsayed H A (2021) Detection of toluene traces in exhaled breath by using a 1D PC as a biomarker for lung cancer diagnosis The European Physical Journal Plus 136:626

[8] Chatterjee S, Castro M and Feller J F (2013) An e-nose made of carbon nanotube based quantum resistive sensors for the detection of eighteen polar/nonpolar VOC biomarkers of lung cancer Journal of Materials Chemistry B 1:4563-75

[9] Moura P C, Raposo M and Vassilenko V (2023) Breath volatile organic compounds (VOCs) as biomarkers for the diagnosis of pathological conditions: A review Biomedical Journal 46:100623

[10] Sun Y, Xia P-F, Korevaar T I M, Mustieles V, Zhang Y, Pan X-F, Wang Y-X and Messerlian C (2021) Relationship between Blood Trihalomethane Concentrations and Serum Thyroid Function Measures in U.S. Adults Environmental Science & Technology 55:14087-94

[11] Fang C, Behr M, Xie F, Lu S, Doret M, Luo H, Yang W, Aldous K, Ding X and Gu J (2008) Mechanism of chloroform-induced renal toxicity: non-involvement of hepatic cytochrome P450-dependent metabolism Toxicology and Applied Pharmacology 227:48-55

[12] Estévez J and Vilanova E 2014 *Encyclopedia of Toxicology (Third Edition),* ed P Wexler (Oxford: Academic Press) pp 885-90

[13] Jo W K, Weisel C P and Lioy P J (1990) Chloroform exposure and the health risk associated with multiple uses of chlorinated tap water Risk Analysis An Official Publication of the Society for Risk Analysis 10:581-5

[14] Kim Y S, Ha S-C, Yang H and Kim Y T (2007) Gas sensor measurement system capable of sampling volatile organic compounds (VOCs) in wide concentration range Sensors and Actuators B: Chemical 122:211-8

[15] V J, Mishra S, Urs K, Tiwary C, Biswas K and Kamble V (2022) Highly Sensitive and Selective Triethylamine Sensing through High-Entropy Alloy (Ti-Zr-Cr-V-Ni) Nanoparticle-Induced Fermi Energy Control of MoS2 Nanosheets ACS Applied Materials & Interfaces 14:
